# Language or rating scales based classifications of emotions: computational analysis of language and alexithymia

**DOI:** 10.1038/s44184-024-00080-z

**Published:** 2024-07-31

**Authors:** Sverker Sikström, Miriam Nicolai, Josephine Ahrendt, Suvi Nevanlinna, Lotta Stille

**Affiliations:** Department of Psychology, Lund University, Lund, Sweden

**Keywords:** Human behaviour, Diagnosis, Information technology

## Abstract

Rating scales are the dominating tool for the quantitative assessment of mental health. They are often believed to have a higher validity than language-based responses, which are the natural way of communicating mental states. Furthermore, it is unclear how difficulties articulating emotions—alexithymia—affect the accuracy of language-based communication of emotions. We investigated whether narratives describing emotional states are more accurately classified by questions-based computational analysis of language (QCLA) compared to commonly used rating scales. Additionally, we examined how this is affected by alexithymia. In Phase 1, participants (*N* = 348) generated narratives describing events related to depression, anxiety, satisfaction, and harmony. In Phase 2, another set of participants summarized the emotions described in the narratives of Phase 1 in five descriptive words and rating scales (PHQ-9, GAD-7, SWLS, and HILS). The words were quantified with a natural language processing model (i.e., LSA) and classified with machine learning (i.e., multinomial regression). The results showed that the language-based responses can be more accurate in classifying the emotional states compared to the rating scales. The degree of alexithymia did not influence the correctness of classification based on words or rating scales, suggesting that QCLA is not sensitive to alexithymia. However, narratives generated by people with high alexithymia were more difficult to classify than those generated by people with low alexithymia. These results suggest that the assessment of mental health may be improved by language-based responses analyzed by computational methods compared to currently used rating scales.

## Introduction

Quantitative assessments of psychiatric conditions are typically based on standardized questionnaires. Major depressive disorder (MDD) and generalized anxiety disorder (GAD) are two leading psychiatric conditions^1^. Improving the screening accuracy of tools is paramount as more accurate and faster assessment is associated with better treatment response and long-term outcomes^2^. Previous research has identified that the inability to recognize and describe one’s own emotions is strongly associated with depression and anxiety^3^. This trait is known as alexithymia, which occurs in ~10% of the general population^4^. Individuals with alexithymia tend to lack mental representations to experience emotions as identifiable and describable feelings, and thus may also experience them to a lesser extent^5^.

Rating scales are well-established assessment tools in current research. MDD and GAD are largely diagnosed and assessed through rating scales, where these scales are based on the DSM-5^6,7^. The employment of these scales is effective as they allow symptoms of psychopathology to be quantified. Perhaps one of the biggest advantages of rating scales is that they allow clinicians to assess specific constructs, as well as the symptoms or perceived causes of a symptom. In general, rating scales can be advantageous as they are easy to utilize, efficient to apply, and simple in their processing and evaluation. However, rating scales also have disadvantages as they require participants to translate their mental states into one-dimensional answers that may not be relevant to the unique and complex mental states of the patients. This raises the question of whether rating scales should be the preferred method for the assessment of mental health^8^.

Expressing feelings and emotions with language may be more intuitive than numeric ratings, as language is the natural way to communicate mental states. Language-based statements are a source of greater inter-individual variation than rating scales. Psychometric properties of rating scales are often challenged, which also applies to scales measuring alexithymia^9^. Therefore, unidimensional surveys may not comprehensively assess the complexity of mental states. Rating scale statements such as “I am sad” may be too rigid and lack sufficient nuance to create an accurate image of one’s feelings and emotions^8^. Following this, language-based measurements may have better validity than rating scales in assessing emotional states, and thus serve as a good addition to current practice.

Verbal language has been shown to allow individuals to communicate their inner thoughts and feelings to others^10^. Similarly, questions based on language measurements permit the assessment of mental states without priming participants with the to-be-measured construct^8^. Recent research has shown that computational models that create multidimensional quantification of texts (e.g., latent semantic analysis, LSA) can be used to assess depression and anxiety, either independently or jointly with rating scales^8^.

Emotional states such as anxiety and depression, or harmony and satisfaction are closely related concepts, which typically show high correlations using rating scales. However, combining the assessment of all four constructs may allow for a more complex assessment of MDD and GAD^8^. By evaluating these closely related concepts together, a fuller picture of an individual’s emotional landscape could be gained. This integrated approach helps in identifying the complex interplay of positive and negative emotional states, enhancing the accuracy of diagnosis and potentially leading to more tailored and effective treatment strategies. Measuring both the negative and positive emotional states provides a more comprehensive assessment of the patient’s emotional functioning than assessing negative states alone. Life satisfaction is strongly associated with the absence of depression and anxiety^11^. Similarly, harmony has a positive effect on depression and anxiety, acting as a buffer^12^. Furthermore, MDD and GAD share high comorbidity, where up to 75% of individuals suffering from a depressive disorder also had lifetime comorbidity of an anxiety disorder^13^. DSM-5 separates MDD and GAD, however, the assessment of MDD also includes symptoms of GAD^14^.

The construct of alexithymia was introduced by Nemiah and Sifneos^15^. People who are high on this trait have deficits in cognitive processing and regulation of emotions^16^, as well as being less organized and differentiated in emotional schemata^17^. This entails a lower ability to make accurate interpretations of their own and others’ emotions. Alexithymia is also connected to a deficit in language expression and reception^16^.

Multiple Code Theory^18^ posits that different types of information, including emotions, are processed using different coding systems. It offers a framework for understanding some of the challenges faced by individuals with alexithymia. Multiple Code Theory suggests that emotional information is one of the types of information that the brain encodes and processes using a specific code. In individuals with alexithymia, there may be a disruption or deficiency in this emotional coding system. This disruption could lead to difficulties in recognizing and labeling emotions, as these individuals may not process emotional information as effectively as those without alexithymia^19^. Language-based assessment of emotional states involves writing a short text about an event connected to an emotional state. As alexithymia relates to individuals’ linguistic abilities, it may lower the accuracy of language-based assessments. Previous research on language-based computational assessment of mental health has successfully shown high validity in a normal population^8^. As this and previous studies have not controlled for alexithymia, there is a risk that participants with high levels of alexithymia may not be a suitable group for computational language-based assessment of mental health. Wotschack and Klann-Delius^20^ investigated alexithymia proficiency using an emotion identification task, which showed that participants with high levels of alexithymia have difficulties in assessing anxiety or depression in themselves and others. Participants with high alexithymia scores produced fewer emotional words and synonyms than participants scoring low on alexithymia^20^. This suggests that alexithymia may be connected to decreased and less varied vocabulary for conceptualizing and expressing emotions and, in turn, make it harder for rating scales to quantify depression or anxiety. However, research shows that the fundamental ability to name emotions might not be compromised in individuals with high levels of alexithymia. One study^21^ indicated that people with high levels of alexithymia were able to name emotions similarly to the control group without alexithymia. This suggests that while experiencing and processing emotional states might be impaired in these individuals, their ability to cognitively label emotions may remain intact. This observation aligns with the Referential Process^19^, which describes the transition from experiencing emotions on a bodily, subsymbolic level to articulating them symbolically through language. While alexithymic individuals may cognitively recognize emotions, they face challenges in connecting these labels to actual emotional experiences and bodily sensations. Additionally, the same study indicated that alexithymic individuals exhibit pronounced difficulties with emotional empathy, a component of emotional processing that demands a deeper understanding and sharing of emotions, as opposed to the more straightforward cognitive empathy tasks like emotion labeling. These findings highlight that although basic emotion naming is not necessarily impaired, the richer language and emotional openness required for nuanced expression and empathetic communication are limited in alexithymia.

Consequently, despite possessing the capability for basic affect labeling, individuals with alexithymia may struggle to express emotions in texts. A literature review found that alexithymia is associated with lower verbal expressiveness in 14 out of 15 studies^10^. Furthermore, they are less open about their emotions compared to participants with low alexithymia scores^22^. Individuals with higher levels of alexithymia tend to rate more extreme emotions, such as anger and fear, as less intense compared to those with lower alexithymia scores. This diminished emotional sensitivity in individuals with high alexithymia levels suggests that they require greater emotional intensity to perceive certain emotions accurately^23,24^. Following the results from previous research, it is unclear if language-based measures apply to individuals with alexithymia.

Previous studies of question-based computational language assessment (QCLA) of narratives have shown a high correlation with rating scales^25^. Here we investigated whether QCLA has higher accuracy in the classification of emotional constructs in narratives compared to rating scales, and to what extent accuracy depends on low or high alexithymia. Based on the previous research, the following hypotheses were formed:

H1: Computational assessment of language-based responses is more accurate in classifying emotional narratives compared to rating scales.

H2: Language-based computational assisted classification of emotional narratives written by participants with high, compared to low, alexithymia scores are more difficult to evaluate.

H3: Language-based computational assisted classification of emotional narratives is less accurate when the words or rating scales assessments are made by participants with high, compared to low, alexithymia scores.

## Results

### Classification

**Fig. 1:**
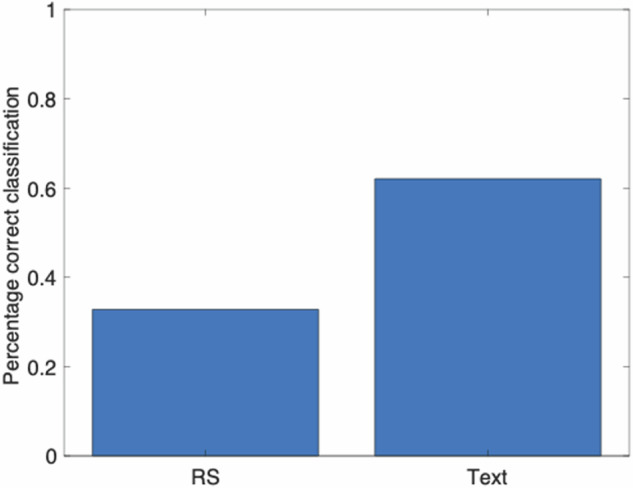
Percentage correct classification based on word response and rating scales in Phase 2. This figure shows the percentage of correct classification for both, semantic measures (text) and rating scales (RS).

The percentage of correct classifications of emotional states based on word responses (62%) was significant (*X*^2^(1, 231) = 19.48, *p* = 0.0000, φ = 0.29), and almost twice as large, compared to when they were based on rating scales (33%, Table 1 and Fig. 1). The baseline probability for correct classification (i.e., the likelihood for guessing) is 25%. Basing the categorization on individual items of the four rating scales (26 items in total, i.e. 9 items for PHQ-9, 7 for GAD-7, and 5 for SWLS and 5 HILS) did not change the categorization accuracy (30%) compared to using the summed scores (*X*^2^(1, 231) = 0.24, *p* = 0.6236, *φ* = 0.03). There was no significant difference between using only word responses compared to combining rating scales and word responses. The percentage of correct classification divided into the four emotions is shown in Table 2.

**Table 1:** Correct categorization in Phase 2 divided into models and low/high PAQ scores in Phases 1 and 2

		Phase 1 PAQ			Phase 2 PAQ		
Trained on	All	Low	High	*P*	Low	High	*P*
RS	0.328	0.387	0.257	0.0368	0.31	0.34	0.6872
Words	0.621	0.681	0.550	0.0434	0.66	0.59	0.3334
Words + RS	0.586	0.630	0.532	0.1344	0.62	0.56	0.4263

The columns in the table show the percentage of correct classifications for all data and for Phases 1 and 2 divided into low and high PAQ scores. The columns labeled *P* show *p*-values for *t*-tests between correction classification of high and low PAQ-scores. The rows show whether the data is classified based on rating scales (RS), words, or words and RS.

*PAQ* Perth alexithymia score, *RS* rating scales.

**Table 2:** Percentage of correct classification based on words divided into emotions for Phase 2

Trained on	Harmony	Satisf.	Depres.	Anxiety
RS	0.23	0.20	0.91	0.00
Words	0.67	0.42	0.75	0.71
Words + RS	0.63	0.35	0.79	0.66

The columns in the table show the percentage of correct classification for harmony, satisfaction, depression, and anxiety in Phase 2. The rows show whether the data is classified based on rating scales (RS), words, or words and RS.

The confusion matrix for the Phase 2 data is found in Table 3. This table shows that the rating scales (in the upper part of the table) have more errors than the word measures in (lower part of the table).

**Table 3:** Confusion matrix in Phase 2

		Harmony	Satisfaction	Depression	Anxiety
RS	Harmony	10	28	2	0
RS	Satisfaction	22	15	1	15
RS	Depression	11	31	51	44
RS	Anxiety	0	0	2	0
Sem	Harmony	29	16	4	0
Sem	Satisfaction	6	31	1	3
Sem	Depression	6	20	42	14
Sem	Anxiety	2	7	9	42

The table shows the confusion matrices between harmony, satisfaction, depression, and anxiety, where the upper part of the table shows the data for the rating scales and the lower for the semantic scales. The columns represent the condition and the rows are categorized by models in Phase 2.

Various classification measures are found in Table 4, including accuracy, precision, specificity, sensitivity, and F1 scores.

**Table 4:** Accuracy, precision, specificity, sensitivity, and F1 scores for rating scales and semantic measures in Phase 2

Data	Measure	Harmony	Satisfaction	Depression	Anxiety
RS	Accuracy	0.73	0.58	0.61	0.74
RS	Precision	0.25	0.28	0.37	0.00
RS	Specificity	0.84	0.76	0.51	0.99
RS	Sensitivity	0.23	0.20	0.91	0.00
RS	F1 score	0.24	0.24	0.53	–
Sem	Accuracy	0.85	0.77	0.77	0.85
Sem	Precision	0.59	0.76	0.51	0.70
Sem	Specificity	0.89	0.94	0.77	0.90
Sem	Sensitivity	0.67	0.42	0.75	0.71
Sem	F1 score	0.63	0.54	0.61	0.71

The table shows accuracy, precision, specificity, sensitivity, and F1 scores for rating scales and semantic measures divided into harmony, satisfaction, depression, and anxiety.

### Test–retest of narratives in Phases 1 and 2

A multiple linear regression was conducted to predict empirical rating scales. The results showed significant Pearson correlations between the empirical ratings and estimated values rating from *r* = 0.33 to *r* = 0.62 (*r*(pred) in Table 5). The Pearson correlation between Phases 1 and 2 estimates based on word responses was higher (0.48 ≤ *r* ≤ 0.77) for all emotions compared to corresponding correlations for rating scales (0.17 ≤ *r* ≤ 0.48).

**Table 5:** Test–retest correlations

	Test–retest			Predicted-ratings		
Construct	*r*(RS)	*p*(RS)	*r*(Words)	*P*(Words)	*r*(pred)	*P*(pred)
PHQ-9	0.47	0.0000	0.77	0.0000	0.62	0.0000
GAD-7	0.19	0.0001	0.48	0.0000	0.33	0.0000
SWLS	0.17	0.0005	0.69	0.0000	0.67	0.0000
HILS	0.48	0.0000	0.77	0.0000	0.47	0.0000

The table shows Pearson correlations between Phase 1 and 2 for rating scales (*r*(RS)) and word response (*r*(Words)) and the probability that the correlations are different than zero given the null hypothesis (*P*). The last two columns show the Pearson correlations (*r*(pred)) between estimated rating scales based on the word response and the rating scales and finally, the probability that the correlations are different than zero given the null hypothesis.

*PHQ-9* Patient Health Questionnaire, *GAD-7* generalized anxiety disorder scale, *SWLS* the satisfaction with life scale, *HILS* harmony in life scale, *RS* rating scales.

### Alexithymia of narrators in Phase 1

All participants were divided into low and high alexithymia based on PAQ scores using a median split (i.e., PAQ ≤ 68 and PAQ > 68, respectively). Participants in Phase 2 had a higher percentage of correct classification when the evaluated narratives were written by participants (in Phase 1) with low compared to high alexithymia. This was true when classification was based on words (68% versus 55% correct classification, *t*(226) = 2.03, *P* = 0.04, two-tailed) or rating scales (39 versus 26% classification, *t*(226) = 2.10, *P* = 0.04, two-tailed) (Table 1).

### Alexithymia of evaluators in Phase 2

Participants with high and low PAQ scores in Phase 2 did not differ in the percentage correct classification. This was not significant using *t*-tests neither when classifications were based on words, nor when it was based on rating scales (Table 1).

### Mean measure of emotions divided into high and low alexithymia scores for Phases 1 and 2

The mean rating scores for each of the four rating scales were significantly higher for the participants with low PAQ scores, compared to those with high PAQ scores; however, no differences were found for the associated language-based estimates of these scores (Table 6).

**Table 6:** Mean ratings and word measures divided into high and low PAQ for Phases 1 and 2

	*m*(RS)			*m*(*W*)		
Emotion	PAQ(low)	PAQ(High)	*P*(RS)	PAQ(low)	PAQ(high)	*P*(*W*)
SWSL	18.31	15.88	0.0024	9.91	10.14	0.6597
HILS	13.50	15.77	0.0153	11.58	11.57	0.9663
GAD-7	15.79	12.74	0.0011	17.87	17.27	0.3384
PHQ-9	8.38	11.24	0.0001	17.30	17.03	0.5557

The table shows the mean score of rating scales (RS) and the associated mean estimated word response scores (*m*(*W*)) for high and low PAQ scores. The *p*-values show whether the low and high scores are significantly different using two-tailed *t*-tests (uncorrected for multiple comparisons).

*PAQ* Perth Alexithymia questionnaire, *PHQ-9* patient health questionnaire, *GAD-7* generalized anxiety disorder scale, *SWLS* the satisfaction with life scale, *HILS* harmony in life scale.

### Standard deviations measure of emotions divided into high and low alexithymia scores

The standard deviations of HILS and GAD-7 were larger for the participants with low PAQ rating scales compared to those with high scores, where the latter finding also was true after Bonferroni correction for multiple comparisons. However, there were no differences in the word estimates of these scores (Table 7).

**Table 7:** Standard deviations of rating and word measures divided into high and low PAQ for Phases 1 and 2

	*s*(RS)			*s*(*W*)		
Emotion	PAQ(low)	PAQ(High)	*P*	PAQ(low)	PAQ(high)	*P*
SWSL	7.58	7.15	0.4509	4.96	4.79	0.6657
HILS	9.30	7.90	0.0330	3.96	3.85	0.7146
GAD-7	9.67	7.25	0.0002	5.98	5.63	0.4348
PHQ-9	6.60	6.79	0.6992	4.35	4.06	0.3697

The table shows the standard deviation of the rating scale scores (*s*(RS)) and the associated estimated word response scores (*s*(*W*)) for high and low PAQ scores. The *P*-values show whether the low and high scores are significantly different using two-tailed *F*-tests (uncorrected for multiple comparisons).

*PAQ* Perth alexithymia questionnaire, *PHQ-9* patient health questionnaire, *GAD-7* generalized anxiety disorder scale, *SWLS* the satisfaction with life scale, *HILS* harmony in life scale.

### Emotional word clouds

**Fig. 2:**
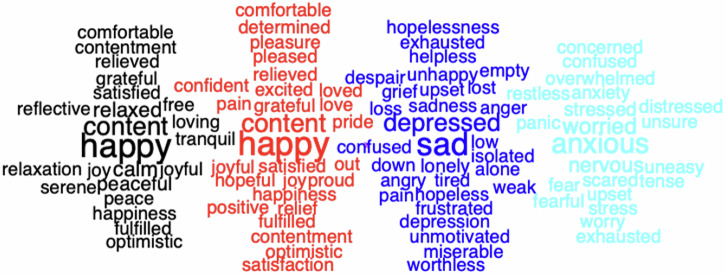
Word clouds for harmony, satisfaction, depression, and anxiety for Phases 1 and 2. Words were classified into the harmony (black), satisfaction (red), depression (dark blue), and anxiety (light blue) categories using the multinomial logistic regression method described in the data analysis section. Each word cloud shows the 25 words with the highest regression coefficients. Font size codes for word frequency in the data set.

Word clouds were generated to describe the words indicative of the four emotional states. Figure 2 shows 25 words with the highest estimates of HILS (black), SWILS (red), PHQ-9 (blue), and GAD-7 (turquoise) scores, respectively. High PHQ-9 scores were associated with *depression*, *sad*, etc., whereas high GAD-7 scores were associated with *anxious, worried, nervous*, etc. Both HILS and SWLS were associated with *happy* and *content*, whereas the former was associated with *calm*, *peaceful*, etc., and the latter with *satisfied*, *grateful*, etc. All aforementioned words are significantly larger than the remaining words on the scales following Bonferroni correction for multiple comparisons. The font size is coded for word frequency in the data set.

### Word clouds of alexithymia and depression

**Fig. 3:**
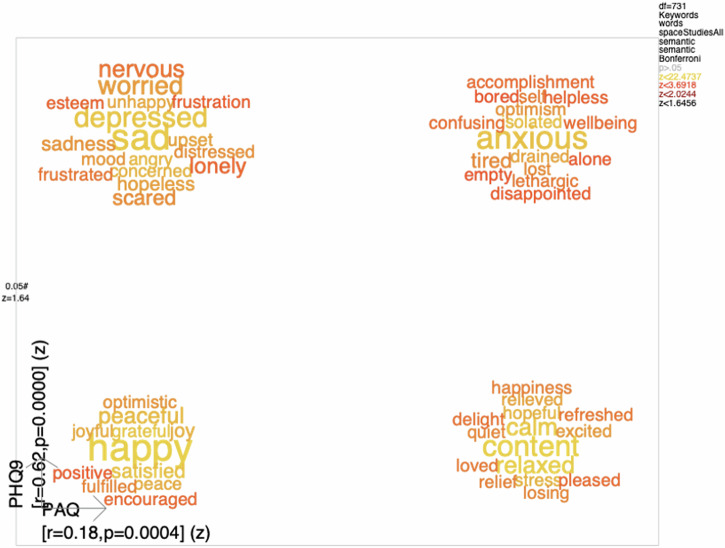
Word clouds associated with low (left) and high (right) PAQ low (down) and high (up) PHQ-9 scores for Phases 1 and 2. See the text for details.

Figure 3 shows four-word clouds where the leftmost are associated with low PAQ scores (≤68) and the rightmost with high PAQ scores (>68). The Pearson correlation between estimated PAQ from words and PAQ rating scale where *r* = 0.18 (*P* < 0.001). The upper clouds are associated with high PHQ-9 scores and the lower with low PHQ-9 scores, where the Pearson correlation between estimated PHQ-9 scores from word response and PHQ-9 scores were *r* = 0.62 (*P* < 0.001).

## Discussion

The present study aimed to investigate whether question-based computational language-based responses or rating scales-based measures show higher validity in classifying emotional narratives and how this relates to alexithymia. The results indicate that language-based responses in emotional narratives can have higher accuracy than rating scales in distinguishing levels of depression, anxiety, satisfaction, and harmony. Participants with high alexithymia scores generated narratives that were more difficult to classify than those with low scores. However, the alexithymia score of the evaluators did not influence the probability of correct classification. The word-based measure had a higher test-retest correlation than the rating scales. Finally, the mean and standard deviation of the alexithymia rating scale scores depended on the alexithymia score, but not on the corresponding semantic estimate.

The present study shows that question-based computational language-based responses more accurately classify the four examined emotional states compared to rating scales. This finding goes beyond the results of Kjell et al.^8^ showed reasonably high correlations to rating scales. Furthermore, Kjell et al.^8^ showed higher validity of computational language-based responses in the classification of emotional states of pictures of faces compared to rating scales, when the emotional states were generated by actors. However, our study is the first to show higher classification accuracy based on language material generated by the participants. Furthermore, the effect size in ref. ^8^ was rather small (i.e., an improvement of 5% where language-based responses showed 83% correct classification compared to 79% for rating scales) compared to the current study (i.e., an improvement of almost 30%). Furthermore, the test–retest correlation between Phases 1 and 2 was considerably larger for language-based responses compared to the rating scales measured for each of the four emotions.

Language-based measures also have other benefits than higher validity and reliability compared to rating scales. Perhaps most importantly, language is the natural way that people communicate their mental states, whereas numerical communication of these states rarely occurs in real-life situations. Empirical data shows that patients prefer communicating their feelings of depression using language as they find it to be more accurate, precise, and natural compared to rating scales^26^. In addition, one aspect where rating scales outperform language is the ease of usage and speed^26^. Furthermore, language-based communication of mental health allows for person-centered care, where the unique situation of patients can be communicated, whereas rating scales are unidimensional and do not allow free expressions.

Another aspect of language-based communication is that it allows for both assessment and treatment of mental health. It is well established that repetitive, expressive writing about self-experienced traumas improves mental health as measured by depression, anxiety, or post-traumatic stress disorder (PTSD)^27^. Combining the expressive writing phenomena with the results from the current study indicates that prompted language-based responses can both assess and treat mental health.

Another aspect of language-based responses is that they can be used to visualize and define mental constructs using word clouds, which is not possible with rating scales. Figure 2 shows indicative words related to narratives of depression, anxiety, satisfaction, and harmony. Depression and anxiety clearly show different word patterns, where these words provide meaningful descriptions or definitions of the two mental states. Furthermore, they also clearly differ from words related to harmony and satisfaction. However, the word clouds for harmony and life satisfaction are rather similar.

The natural language processing methods have recently been significantly improved by deep neural network models that are enhanced by attention mechanisms called transformers. For example, bidirectional encoder representations from transformers (BERT)^28^, which is perhaps the most widely cited transformer-based language model, has shown great performance in a number of language tasks. However, the main advantage of transformer-based models is that they are able to understand the grammatical context that is in free texts, whereas the current data is based on context-free descriptive words where non-contextual models such as LSA perform well. The current data was also analyzed using BERT, however, although the results were similar, it did not improve over the used LSA model.

The alexithymia scores did not influence how accurately the participants classified the emotions, neither based on rating scales nor on language-based responses. These results uphold the notion that simply being able to identify emotions by name does not automatically negate the possibility of alexithymia, since the disorder encompasses a deeper challenge in emotional consciousness and engagement beyond mere verbal identification^21^.

However, our results found that the participants with high alexithymia scores generated narratives that were more difficult to classify than those of the participants with low scores. This was true both when the classification was based on rating scales and on word responses. This is also in line with previous research showing that individuals with alexithymia often struggle with the Referential Process, which involves translating subsymbolic emotional experiences into symbolic, linguistic representations. According to Multiple Code Theory, this difficulty arises because those with alexithymia may not efficiently bridge the gap between the bodily, affective experience of emotions and their cognitive, verbal expression. Consequently, their emotional narratives lack the richness and clarity seen in individuals without alexithymia, making their emotional language more ambiguous and harder to categorize using standard linguistic and psychological tools^19^.

These results are consistent with previous findings showing that alexithymia is a deficit in language expression^16^. However, our results do not support the previous findings that imply that individuals with alexithymia have difficulties taking the perspective of another person, which in turn would make the identification of other people’s emotions in the text more difficult^29^. found that individuals with alexithymia did not show more narrative engagement when reading a first-person story in comparison to a third-person story. However, individuals with low alexithymia scores showed more narrative engagement^29^. The authors argued that the reason for this effect is that individuals with alexithymia have difficulties in mentally simulating another person’s perspective. Alexithymia was not found to have an effect on judging someone else’s emotional text written in the first-person perspective in our study.

Furthermore, alexithymia is linked to mentalizing deficits, which are related to the inability to imagine other people’s mental states^30^. Therefore, it was expected that participants with high alexithymia scores provide significantly lower classification accuracy in the word measure than participants with low scores, which was not found in our data. The difference in correct predictions for alexithymia related to the participants generating narratives versus evaluating the narratives may be understood by the fact that it is more difficult to generate than evaluate emotional narratives^20^.

A limitation of the study is that it was conducted in a normal population. A pre-screening with a selection of participants with alexithymia scores above certain criteria may have generated stronger effects. Nevertheless, the prevalence of being diagnosed with alexithymia in the general population is ~10%^4^.

Additionally, self-reports were used as a measure of alexithymia. While they may have biases, especially in self-evaluative capacities, which can be limited in those with alexithymia, they still offer valuable information on how people view their emotional abilities and challenges^31^. Using self-reports also allows for consistency with existing literature, facilitating comparison and contributing to the broader understanding of alexithymia^9^. These are practical for large-scale data collection and are well-validated in psychological research, making them suitable for studying alexithymia. They provide crucial insights into individuals’ self-perceived emotional experiences, which is especially relevant for alexithymia, characterized by difficulties in processing and communicating emotions. Despite potential limitations in capturing the full spectrum of emotional awareness, self-report data remain a useful and important aspect of psychological research on emotional processing disorders.

Furthermore, the study was conducted online, leaving less possibility to monitor and control how the participants conducted the task. To minimize this effect, we used control questions to detect participants who did not follow the instructions.

Finally, perhaps the biggest limitation is that the rating scales in Phase 2 were not filled in by multiple people for each text. This means that the inter-rater reliability cannot be assessed, and thus the overall reliability of the method cannot be fully assessed. In addition, the texts were not rated by clinicians, and further research would benefit from assessing the inter-rater reliability both on a general level as well as between the general population and clinicians.

Overall, as our study did not include clinicians rating the texts, the results of our study can not be generalized. It is likely that clinicians who have had comprehensive training in psychopathological symptoms would have more success in rating the states correctly. Future research should aim to include clinicians for this reason as well.

Finally, it is likely that constructs that are incorporated into rating scales are constructs that very rarely come up in a spontaneous text. For example, anhedonia, while a central symptom of depression, is not something that one thinks to report when asked to write about their experience of depression. This means that the use of free text may not catch all symptoms of depression and anxiety. Therefore, the use of free text in this form should be limited to the triage of psychopathology at this stage, as it can not replace the use of rating scales by a qualified clinician.

The possibility of improving the assessment of mental health with language-based research has great potential. Future research should aim at a computational-based assessment of language to improve the evaluation of mental health. More accurate, earlier, and reliable measures of mental health may allow more specific treatment and increase the possibility of recovery and shorter sick leaves. Future research should also aim at evaluating whether computational language-based methods are more accurate in the assessment of depression and anxiety according to the DSM-5 criteria in clinical settings. Language-based measurements could be compared to other methods than self-report measures, such as psychological interviews to improve the diagnostic process. Narratives generated for specific emotional states could offer insight into less common manifestations of depression and anxiety, which are missed when using traditional methods. The research into semantic methods is nascent and its generalizability to different groups should be further examined.

Future research may investigate the validity of language-based responses in measuring other psychological constructs than depression, anxiety, satisfaction, and harmony. For instance, an interesting question would be if language-based responses may improve the validity of measuring the BIG-5 personality traits. Another possibility is to investigate whether language-based models are a valid method for the assessment of other complex disorders, such as personality disorders.

The present study indicates that a computational-based assessment of classifying emotions based on word responses can be more accurate than classifications based on rating scales. The classification accuracy did not depend on the alexithymia scores, however, people with high alexithymia scores generated narratives that were more difficult to classify. This suggests that computational assessment of language may be a valid method for classifying emotional states that are also applicable to individuals with alexithymia. Future research should investigate whether computationally assisted language-based response improves the accuracy of assessment of mental health in clinical settings.

## Methods

### Transparency and openness

We report how we determined our sample size, all data exclusions, and all measures in the study, and we follow JARS^32^. Data were analyzed using the online software Semantic Excel (semanticexcel.com; see also ref. ^33^) and IBM SPSS Statistics for Windows, Version 27. This study’s design and its analysis were not pre-registered.

### Participants

The inclusion criteria were adult US residents with English as their first language and an age of 18 years or older. Participants gave written consent to participate in the study, and were excluded from the analysis if they did not consent, did not complete the entire survey, or answered the control questions incorrectly. They were also excluded if they did not follow the instructions in their free text or descriptive word responses. For example, the text contained only a single sentence or did not indicate one of the four emotional states (e.g. “Anxiety is a worthless emotion.” “I don’t feel anxiety.”). Based on these criteria, 34 of a total of 150 participants were excluded in Phase 1. In Phase 2, after excluding 18 of 250 participants, 232 participants were included in the analysis.

Of the participants included in the analysis in Phase 1, 78 were female, 31 were male, five were nonbinary and two preferred not to say. The age range was from 20 to 73 years (*M* = 39.7, SD = 13.9). In Phase 2, there were 150 females, 73 males, seven nonbinary, and two preferred not to say. The age range was from 18 to 79 years (*M* = 32.66, SD = 11.15).

### Materials

Symptoms of anxiety and depression were assessed with the Generalized Anxiety Disorder scale (GAD-7)^34^ with seven items such as “How often in the past two months have you felt nervous, anxious or on edge?” (*α* = 0.93) and the Patient Health Questionnaire (PHQ-9)^6^ with nine items such as “How often in the past two months have you been bothered by little pleasure or interest in doing things?” (*α* = 0.90). Both used a scale from *not at all* (0), *several days* (1), *more than half the days* (2), and *nearly every day* (3).

For measuring harmony, the Harmony in Life Scale (HILS^25^); was used. It uses five items, such as “My lifestyle allows me to be in harmony” (*α* = 0.94). The Satisfaction with Life Scale (SWLS)^35^; contained five items, such as “In most ways, my life is close to my ideal” (*α* = 0.93).

For the assessment of alexithymia, the Perth Alexithymia Questionnaire (PAQ)^17^; was used. It consists of 24 items with questions such as “I tend to ignore how I feel” (*α* = 0.96). The PAQ, HILS, and SWLS were all rated on a 7-point Likert scale ranging from *strongly disagree* (1) to *strongly agree* (7).

The narrative participants were required to compose a narrative about a recent experience of depression/anxiety, satisfaction/harmony within the last two months in Phase 1 (~5 sentences) was utilized as material in Phase 2 to be evaluated by another set of participants. All participants were asked to write 5 descriptive words indicating the emotional state described in the narrative (see the supplementary information for the exact instructions). The semantic questions for depression, anxiety, life satisfaction, and harmony in life have been developed and validated in the general population by Kjell et al.^8^. The exact phrasing of these questions can be found in Supplementary Information.

### Procedure

Participants were recruited using Prolific for a compensation of £2 for Phase 1 and £1.25 for Phase 2 (based on a rate of £6/h). The study was conducted in English. Every participant gave consent at the beginning of the study. The survey was published on Qualtrics and was accessed via a link.

The study consisted of two phases. During Phase 1, the participants were asked to write one autobiographical text (~5 sentences) about a period of their life within the last two months during which they experienced one of the four emotional states harmony, satisfaction, depression, or anxiety. They were randomly assigned to one emotional state and instructed not to include the exact word of the emotional state in their narrative (i.e., not to write harmony, satisfaction, depression, anxiety). Participants were informed that the text they wrote would later be read by other participants. Thereafter, each participant was asked to write down five words describing the same emotional state as in their text. Additionally, they were instructed to fill out the rating scales HILS, SWLS, GAD-7, PHQ-9, and PAQ based on the emotions from the narrative. Finally, they were asked about their demographic information. Phase 1 took ~20 min to complete.

During Phase 2, another set of participants first read one of the texts about an emotional state composed by a participant from Phase 1. Then, participants were asked to describe the emotional state of the narratives using five descriptive words. Afterward, they filled out the same rating scales as stated in Phase 1 based on the text they had read to assess the author’s emotional state. Phase 2 took approximately 12 min to complete.

### Data analysis

#### Creation of semantic representations

The descriptive words generated by the participants were quantified using latent semantic analysis (LSA), following the procedure by Kjell et al.^8^ (see also ref. ^36^). LSA generates a high-dimensional representation of words based on how they co-occur in a corpus. We selected a database consisting of words generated by participants in experimental conditions related to the five-word generation task in the current experiments (e.g. “Describe whether you are depressed/anxious/satisfied/in harmony with three/five/ten descriptive words) taken largely from Kjell et al.^8^, but also other related publications. This dataset consisted of 69,167 words in total and 6630 unique words generated from 7088 responses to open-ended questions.

Based on this database, a word-by-word co-occurrence frequency table was generated, where the contexts were the word responses for participants from a given question. The cells in the table were normalized by taking the logarithmic plus one. A data compression algorithm called singular value decomposition (SVD) was applied to this table, where the dimensions are ordered after the accounted variance in the original matrix. The first 300 dimensions were used. This resulted in a semantic representation where each word is represented in a high dimensional vector, which was normalized to the length of one.

A semantic representation of the five words used to describe the narratives in the current study was generated by adding the associated vector in the semantic representation and normalizing the length of the resulting vector to one.

#### Classifying the emotions of the narratives

Multinomial logistic regression was used to classify the participants’ responses into one of the four possible emotions. The word responses were based on the semantic representation of the five words. The rating scales were based on the total scores of the four rating scales. In addition, we also made a model based on the combination of the total scores of the rating scales and the semantic representations. The multinomial logistic regression was evaluated by a 10% nested cross-validation leave-out procedure^37^. A model was generated based on 90% of the data and then evaluated by the left out 10% data. To avoid overfitting, the grouping was made so that all responses to a specific narrative were always either in the train or the test datasets. This procedure was repeated ten times so that classification was made on all the data points. In each training fold, a nested cross-validation procedure was used, where the number of semantic dimensions used was optimized, so that the mean number of first dimensions was 33.8 with a standard deviation of 6.5. To increase the size of the training dataset, we included data from a related study with the same number of participants (*N* = 348)^38^, that used the same procedure as in this article; however, the data from that study did not include a measure of alexithymia. The training was conducted on data from Phase 1 and Phase 2, but the evaluation of the correct classification was only done on the Phase 2 data. Thus, the size of the training dataset was *N* = 732, and where this article applies and presents the results of the data, including alexithymia with *N* = 348.

Word clouds were generated by the same multinomial logistic regression as described above. However, here the semantic representation was based on individual words.

Multiple linear regression was also conducted to predict empirical rating scales, where the number of dimensions was optimized using the same cross-validation and leave-out methods as the multinomial logistic regression.

### Supplementary information

Supplementary Information

## Data Availability

All data have been made publicly available at the Open Science Framework and can be accessed at https://osf.io/7ukf9/.

## Code availability

The analysis conducted in this paper can be done in the online software Semantic Excel (www.semanticexcel.com; see also ref. ^33^). A high-level code can also be found at https://osf.io/7ukf9/.
