# Supplementary material for: Language or rating scales based classifications of emotions: computational analysis of language and alexithymia
Source: Npj Ment Health Res. 2024 Jul 31;3:37. doi: 10.1038/s44184-024-00080-z (PMC11291691; doi:10.1038/s44184-024-00080-z)
Supplement: Supplementary file 1 — Supplementary Information [file 44184_2024_80_MOESM1_ESM.pdf]

Supplementary Information

Supplementary Methods

Semantic question in Phase 1. Please write a short text about when you have experienced (harmony/satisfaction/anxiety/depression) recently (within the last 2 months). Please answer the question by writing at least a paragraph (approx. 5 sentences) below that indicates whether you are in (harmony/satisfaction/anxiety/depression) or not. Try to weigh the strength and the number of aspects that describe if you are in harmony or not so that they reflect your overall personal state of (harmony/satisfaction/anxiety/depression). For example, if you are in harmony then write more about aspects describing this, and if you are not in harmony then write more about aspects describing that. Write about those aspects that are most important and meaningful to you.

Descriptive word questions in Phase 1 and 2. In Phase the semantic question was phrased as "Please answer the question by writing 5 descriptive words below that indicate the emotional state you described in the text." In Phase 2 the semantic questions the question was phrased as "Please write 5 words about the emotional state you believe the author was describing in the text." In both Phase 1 and 2 this was followed by: "That is, think about the text you wrote before, and try to write the words about the same event. Try to weigh the strength and the number of words that reflect your overall personal state of mind. For example, if you are feeling (harmony/satisfaction/anxiety/depression) then write more and stronger words describing this, and if you are not (harmony/satisfaction/anxiety/depression) then write more and stronger words describing that. Write descriptive words relating to those aspects that are most important and meaningful to you. Write only one descriptive word in each box. Please do not use the word that was used as a prompt for the text you wrote. Please answer the question by writing 5 words that describe your emotional state. Write only one descriptive word in each box."
